# Oral Contact Events and Caregiver Hand Hygiene: Implications for Fecal-Oral Exposure to Enteric Pathogens among Infants 3–9 Months Living in Informal, Peri-Urban Communities in Kisumu, Kenya

**DOI:** 10.3390/ijerph15020192

**Published:** 2018-01-24

**Authors:** Emily Davis, Oliver Cumming, Rose Evalyne Aseyo, Damaris Nelima Muganda, Kelly K. Baker, Jane Mumma, Robert Dreibelbis

**Affiliations:** 1 Department of International Health—Social and Behavioral Interventions, Johns Hopkins Bloomberg School of Public Health, Baltimore, MD 21205, USA; emilydavis616@gmail.com; 2 Department of Disease Control, London School of Hygiene and Tropical Medicine, London WC1E 7HT, UK; Oliver.Cumming@lshtm.ac.uk; 3 Department of Community Health and Development, Great Lakes University of Kisumu, Kisumu 40100, Kenya; evalyneaseyo6@gmail.com (R.E.A.); damarisnelima@yahoo.com (D.N.M.); 4 Department of Occupational and Environmental Health, College of Public Health, University of Iowa, Iowa City, IA 52333, USA; kelly-k-baker@uiowa.edu; 5 Nutrition Department, School of Public Health, Great Lakes University of Kisumu, Kisumu 40100, Kenya; janemumma@gmail.com

**Keywords:** pathogen exposure, caregiver handwashing, direct observation, enteric infection, childhood diarrhea

## Abstract

Childhood diarrhea is one of the leading causes of morbidity and mortality in children under five in low and middle-income countries, second only to respiratory illness. The mouthing behavior that is common in children exposes them to fecal-orally transmitted pathogens that can result in diarrhea; however, there is a need for further evidence on specific exposure routes. This study describes the frequency and diversity of two important routes of enteric pathogen exposure among infants 3–9 months of age: infant oral contact behavior and caregiver handwashing behavior. Data were collected through structured observations of 25 index infants for the oral contact data and 25 households for the caregiver handwashing data in a peri-urban setting in Kisumu (Obunga), Kenya. Breast was the most common type of oral contact event with an average of 3.00 per observation period and 0.5 events per hour. This was followed by a range of physical objects with an average of 2.49 per observation and 0.4 events per hour. The “infant’s own hands” was the third most common oral contact, with an average of 2.16 events per hour, and 0.4 oral contact events per hour. Food and liquids were the 4th and 5th most common oral contact events with an average of 1.64 food contacts and 0.52 liquid oral contact events per observation period. Feeding events, including breastfeeding, were the most commonly observed key juncture—71% of total junctures observed were caregivers feeding children. This was followed by child cleaning (23%), caregiver toilet uses at (4%), and lastly food preparation at 2%. HWWS was observed only once before a feeding event (1%), twice after cleaning a child (9%), and twice after caregiver toilet use (40%). The combined implication of data from observing oral contact behavior in children and hand hygiene of caregivers suggests that caregiver hand hygiene prior to feeding events and after cleaning a child are priority interventions.

## 1. Introduction

Diarrhea remains one of the leading causes of morbidity and mortality for children under the age of five in low-income countries. Recent estimates suggest that over 2000 children die every day from diarrhea-related illness as a result of enteric infection, accounting for 1 in 9 child deaths globally [1]. However, both symptomatic and asymptomatic enteric infection can have negative impacts on child health, such as [2] growth faltering and reduced cognitive development [2,3]. In low income settings, the burden of both enteric infection [4] and diarrhea [5] remains high even in the first year of life when the process of stunting begins and can accelerate dramatically [6]. A stronger understanding of the dominant environmental exposure pathways for enteric pathogen infections pathogens during early childhood and infancy, particularly with regard to water, sanitation and hygiene, may contribute to more effective interventions [7]. 

Improvement of water, sanitation, and hygiene (WASH) in communities with high prevalence of enteric infection and the continued promotion of breastfeeding are critical to reducing the prevalence of diarrhea [5,8,9]. Oral exposure to pathogens is a basic tenet of the fecal-oral transmission route associated with enteric infections. Past studies on the oral contact behavior—oral contact with any object, surface, liquid, or body part (own or other)—of children have widely varied in terms of methodology, geographic location, age of observed children, and the definition of oral contact or “mouthing”. Past studies quantifying child contact with environmental hazards in children in low income settings have focused on exposures or hazards, including: objects [10,11], pesticides [12], and bacterial indicators of animal and human feces via hands, objects, or drinking water [13,14]. In a 2007 meta-analysis, Xue and colleagues found that there was a lack of oral contact data for infants aged 0–6 months [15]. Hand hygiene is widely researched, with the majority of studies focusing on the motivations, duration, practice, and habitual nature of handwashing. However, these studies lack oral contact information that would provide a comprehensive view of exposure pathways for enteric pathogens [16,17]. Further, most studies like those above have focused on handwashing practices among caregivers of children from a wide age range and were conducted in rural settings rather than in low-income urban or peri-urban areas. Research is lacking on child exposures in low income urban settings where high population density combined with limited public health infrastructure can create highly contaminated environments [18]. 

This study addresses these gaps by assessing caregiver hand hygiene and the frequency and type of oral contact events among infants aged 3–9 months at the household level in a peri-urban slum of Kisumu, Kenya. The household setting was chosen as multiple studies in different countries have shown that fecal pathogen exposure can be reduced through interventions targeting behaviors specific to the household domain, including improved hand hygiene practices and increased and improved child food preparation practices [19,20,21]. 

The aim of this study is to better understand how an informed intervention could reduce the child and caregiver behaviors that can result in pathogen fecal-oral pathogen exposure and infection among children 3 to 9 months of age. The specific objectives were to observe infant (ages 3 months to 9 months) oral contact behavior, caregiver hand hygiene practices, and other related actions to identify the environmental, social, and habitual factors that contribute to enteric pathogen exposure in urban Kisumu. Exposure and behavioral data reported here contributed to the development of a comprehensive intervention for reducing early life exposures to enteric pathogens.

## 2. Materials and Methods 

A mixed method, cross-sectional observational study was completed assessing enteric pathogen exposure routes—specifically oral contact events—among children 3–9 months of age and caregiver hand hygiene practices. Ethical approval for this study was granted by the Ethics Committee of the London School of Hygiene and Tropical Medicine (Ref. Number: 11717) and the National Council for Science, Technology, and Innovation (“NACOSTI”) (Ref. Number: GREC/010/248/2016). Written consent to participate was provided by all participating caregivers in advance of any data collection. The data collection team was trained on the ethical procedures for the study including how to obtain written informed consent from study participants, confidentiality, and data management practices. The study was conducted in accordance with the Declaration of Helsinki. 

### 2.1. Study Setting

The study was conducted in a low-income informal settlement in Kisumu, Kenya. The study site is a part of a larger peri-urban informal settlement belt that encircles the more developed urban core of Kisumu. Approximately 60% of Kisumu residents live in peri-urban settlements surrounding the city [22]. Access to safe drinking water and sanitation in the study area is limited; most inhabitants rely on paid, shared water sources and rudimentary pit latrines shared by multiple households. In 2015, a cross-sectional survey in the study area found high levels of contamination with fecal pathogens in household-stored drinking water and household prepared food (unpublished). 

### 2.2. Sampling 

The observational data were collected from a purposive sample of households in the study area. All participating households met the following criteria: (1) located in the defined study area; (2) have a residing child between 3 and 9 months of age; and, (3) have a consenting adult present at the time of observation. Community Health Volunteers (CHVs), who serve as the link between the household system and the county health system by providing health information and referrals to families in their community, helped the research team identify all households in their catchment areas that met the eligibility criteria. In addition to identifying households, the CHVs provided an initial explanation of the study prior to the formal consent process conducted by the study enumerator. 

### 2.3. Data Collection

#### 2.3.1. Oral Contact Events

Oral contact data were collected through structured observations in the household setting. In sampled households, an index child between 3 and 9 months of age was selected as the focus of observation. Oral contact events were defined as any time an object, food or otherwise, came into contact with the child’s mouth either in the form of mouthing/gumming or for the purpose of ingestion. Objects included the index child’s own hand, others’ hand, foods, liquids, objects, clothing, dirt or soil, furniture, or components of the household structure itself. Observations lasted up to 6 h and were conducted during either the morning hours (07:00–13:00) or during the afternoon hours (13:00–20:00). 

The index child was verified as meeting the eligibility criteria at the beginning of the observation period by the CHV and enumerator through confirmation of the child’s age as noted on the clinic card or verbally confirmed in the absence of the clinic card. There was only one child per observation. During observation, the enumerator recorded any object that entered or came in contact with the child’s mouth, the perceived level of contamination of the object, changes in the index child’s primary activity, any caregiver action taken to mitigate to oral contact events, changes to the index child’s location or environment, and contact with a caregiver. Object contamination was defined as a categorical indicator based on visible contamination with dirt or other matter (yes/no/unknown). Caregiver contact was defined as any time the index child came into direct contact with the caregiver (point of contact yes/no). A mitigation event was defined as any time the caregiver performed an action that sought to reduce the index child’s pathogen exposure, and included action such as removing a potentially contaminated object from the index child’s possession, washing the mouthed objects and returning it to the index child to continue to be mouthed, or replacing a contaminated object with a clean object. Location or environmental change recorded any time the child was picked up, moved to a different area of the household, taken outside, or taken to a different household. Data were collected to assess how changes in environment could potentially impact the type of oral contact behavior of the index child. 

#### 2.3.2. Caregiver Handwashing 

The caregiver hand hygiene data collection consisted of structured observations in households. These caregiver observations were conducted during the morning (07:00–13:00) or during the afternoon (13:00–20:00). In each household, an index child was specified and observations focused on the index child’s primary caregiver—the individual responsible for child care at that moment in time. If the primary caregiver changed to a different individual, this was recorded and the new primary caregiver was observed. The primary indicators for these observations were: handwashing behaviors in relation to sanitation/and toileting, child feeding, child cleaning, and food preparation. These four key “junctures” were chosen based on a qualitative analysis of the index child observation data and expanded field notes as well the existing literature, which point to these events as the most likely to result in child pathogen exposure [20,21,23]. The response categories for hand hygiene were: hand washing with water only, washing with soap and water, or no handwashing in relation to the key junctures. In addition to hand hygiene, the enumerator also recorded activity or location changes among all potential caregivers in the household. Observers recorded when the caregiver left, returned, had contact with another adult, or became the primary caregiver in the circumstances of transfer of caregiver responsibilities. While recording this “phase or location change” of a caregiver the enumerator would also note details about the relation of the caregiver to the child in a roster. 

All observational data were recorded on mobile phones (Alcatel, Pixi 3, Gigaset AG, Munich, Germany) using structured observation tools developed with Open Data Kit (ODK). All data were sent to the ODK aggregate immediately after collection and reviewed for accuracy by the study team. Further analysis of the data was conducted using STATA version 14 (StataCorp LP., College Station, TX, USA). 

#### 2.3.3. Expanded Field Notes and Daily Debriefing Session 

During data collection, enumerators wrote expanded field notes containing any relevant information that was not expressly requested in data collection tools. This additional information included details regarding the visible health of the index child, the disposition of the mother and child, the caregiver’s profession or source of income, the location of the latrine, the state of the latrine, the perceived economic status of the household, and other details that may impact the mouthing behavior of the child or the hand hygiene of the caregiver. The expanded field notes and the data collected using the ODK observation tool were discussed after each day of data collection with the research manager. Daily debriefing sessions became a critically important opportunity for troubleshooting issues encountered during data collection and to ensure that the enumerators were adhering to ideal data collection practices. Much of the qualitative data pulled from the expanded field notes shed light on the overall findings of the study.

### 2.4. Analysis

Data were exported from the ODK aggregate to STATA IC (StataCorp LP., College Station, TX, USA) for analysis. Two datasets were generated—one for oral contact events and one for caregiver hand hygiene events. Summary statistics were calculated from the data and included total observation time, average number of observations per data collection period, average number of observations per hour, age and sex distribution of participants, and the total number of observations per key indicator (Table 1).

The oral contact events dataset was analyzed to determine which objects enter the child’s mouth the most throughout the observation period. The analysis assessed if oral contact behavior differed by sex or age of the child, by location (indoor, outdoor) of the event, and by the time of day the event occurred. The caregiver hand hygiene data were analyzed with a focus on how often handwashing was completed at the appropriate time in association with key junctures (points in time when child exposure to dangerous pathogens is most likely to occur e.g., before food preparation, before/during child feeding, or after child toileting). By analyzing the total number and frequency of observations, both child exposure, and caregiver hand hygiene events, the study team was able to assess event by age and sex. To accommodate an unequal skew in ages and sex the data was weighted by time observed. Because observations were reviewed daily and data discussed and reviewed on an on-going basis, quantitative measures of inter-observer variability are not provided.

A qualitative analysis of the expanded field notes and daily debriefing notes collected by enumerators was also performed. A coding system was created to identify major recurrent themes, barriers to ideal behaviors, and contextual factors using an emergent coding process whereby codes were organically created and iteratively assigned to data points identified in the expanded field notes and daily debriefing notes. After the initial coding, a secondary coding was conducted on the same data set by another member of the data analysis team. This was to assess inter-rater reliability. When assessing the two versions of coding it was determined that the coding was approximately the same between different coders. 

## 3. Results

Table 1 and Table 2 presents descriptive statistics for the structured observations, including the number of observations performed, the average observation time, and the sex and age distribution for index participants for each observation. 

All observed infants were visibly healthy at the time of observation. Data about the specific health status of the child or caregivers were not collected.

### 3.1. Oral Contact Events 

A total of 264 oral contact events were observed during 142 h of observation spanning 25 infants, with an average of 1.76 contact events per child per hour. There was mean of 10.6 oral contact events per observation period, equivalent to 1.8 oral contact events per hour of observation. Breast was the most common type of oral contact event with mean of 3.00 per observation period and 0.5 events per hour. This was followed by a range of physical objects—including toys, clothing items, dirt, phones, and keys (mean: 2.49 per observation; 0.4 events per hour). The “infant’s own hands” was the third most common oral contact (mean: 2.16 per observation; 0.4 per hour) Food and liquids were the 4th and 5th most common oral contact events (Table 3). Children were almost exclusively fed cooked foods—such as porridge, potatoes, or ugali—while feeding of raw foods was extremely rare.

Approximately 28% of all oral contact events were with the breast; objects accounted for another 24% of oral contact events; and the child’s own hands an additional 20%. Food and liquids combined accounted for 21% of all oral contact events observed (foods: 16%, liquids: 5%). Children between the ages of 3–6 months were responsible for a weighted 53% of oral contact events (Table 4). 

Differences in oral contact events were also observed by child age. Children between the ages of 3–6 months were more likely to have more objects, food, liquids, and other oral contact events. Children between the ages 7–9 months were more likely to have more breast, their own hands, and the hands of others as oral contact events (Table 4).

### 3.2. Caregiver Hand Hygiene

A total of 101 critical junctures were observed during 138 h of caregiver observation. Feeding events, including breastfeeding, were the most commonly observed key juncture—71% of total junctures observed were caregivers feeding children. This was followed by child cleaning (23%), caregiver toilet uses at (4%), and lastly food preparation at 2% (Table 5).

Handwashing with soap (HWWS) was observed 5 out of 101 times at the appropriate juncture associated with a possible caregiver contamination event (Table 6). HWWS was observed only once before a feeding event (1%) and twice after cleaning a child (100%). Handwashing was observed twice after toilet use (100%). HWWS was least common before food preparation. There was only one observed instance of caregivers rinsing hands with water without using soap. From a public health perspective, handwashing was noted out of sequence with practices that would reduce risk; such as washing hands after feeding a child or after preparing food. Observed events often occurred in rapid sequence and sequences of events often recorded as one action. The majority of these instancess were resolved in debriefing; however, a limited number of events remained that could not be resolved. This included a total of 9 times that hands were washed with soap; however, the proper sequence (i.e., before feeding; after toilet use) could not be determined. 

## 4. Discussion

This study provides data on the oral contact behavior and handwashing practices of primary caregivers; highlighting potential routes of exposure to enteric pathogens in infants between 3 and 9 months of age. Frequencies of different types of oral contact events varied between younger versus older children. This finding was also seen in other studies that assessed oral contact frequency although the age range of observed children was slightly higher than that of this study [12,24]. For example, consumption of food or water was 2 to 4 times more common in younger infants compared to older ones. One potential explanation for this difference is that feeding frequency reduces as infants transition from an all-liquid breast milk diet to heavier solid foods, and the small study sample size of children (*n* = 25) was insufficient to capture increasingly infrequent daily child feeding practices. Timing observation to capture relatively rare (few times per day versus few times per hour) exposure events is challenging, and the accuracy of measurements can be heavily skewed when observation periods are limited to 4 to 6 h blocks of time, per common practice [13,25]. 

The infant’s own hands was the second most common type of oral contact after breast. Specific data regarding child handwashing during the child cleaning process was not specified. This finding is in keeping with results from a study in Tanzania in which child’s hands were the second most commonly mouthed object, second only to food [3]. In future data collection around child cleaning, child handwashing should be represented by its own set of indicators as an important intervention point to reduce child exposure to pathogens. Breast comprised 28% of all oral contact events. The extent to which breast contact was observed is encouraging. The World Health Organization has recommended exclusive breastfeeding for the first six months of life and complementary breastfeeding for the first two years [26]. This recommendation is the foundation of many infant and young child feeding campaigns as is widely promoted throughout the developing world. A similar study observed breast contact in only 18% of all oral contacts events, however the study examined a larger age range of children. Instead, the authors found a high proportion of oral contact events due to the child’s own hands (87%)—similar to the results of our study [3]. In several previous studies in different populations, pacifiers were the primary mouthed object in both frequency and duration. Infants that are provided pacifiers may be less likely to mouth other objects [27]. In one study pacifiers were intentionally excluded from analysis in order to reduce the impact that it would have on study findings [11]. The variety of objects mouthed by infants in this study population could potentially be more varied due to the lack of available pacifiers. The range of objects observed to enter children’s mouths is more likely to result in erratic and unpredictable oral contact patterns compared to food, liquids, and breast.

Material objects that comprised the majority of oral contact events are likely to be handled by caregivers in a random manner. Specific, targeted interventions to reduce contamination and infant exposures to enteric pathogens may be difficult due to the stochastic nature of both oral and hand contact. This reinforces the importance of HWWS as a primary barrier to pathogen transmission within this context. During all observations, HWWS was observed at only 5% of key junctures, below global averages for HWWS [28]. When participants did perform a hand hygiene behavior they used soap and water in most instances of the time. Hand rinsing with water only was only observed in association with one key juncture, food preparation. These findings suggest initiation of handwashing is a critical health intervention rather than focusing on handwashing technique and practice. 

Food preparation was never proceeded by HWWS and only observed before 1% of feeding events. Both preparation and feeding are a part of a larger system and feeding typically follows preparation in close succession. Therefore, caregivers may assume that handwashing prior to feeding is unnecessary if handwashing prior to food preparation was practiced. The importance of handwashing in the behavior system focused on food is an important exposure pathway—it is the third most common type of oral contact withstanding breast. The study by Nizame et al. also reflected the importance of hand hygiene during the larger food behavior system. Authors recommended that improved access to handwashing materials such as running water and soap in conjunction with targeting executive motivations is the best way to increase handwashing around both meal preparation and feeding events [17]. Our findings also reinforce that health messaging must target the importance for handwashing prior to meal preparation and before feeding. 

Caregiver handwashing after child cleaning was observed after 40% of child cleaning observations. In similar studies handwashing after child cleaning was observed 32% the time and 47% respectively [3,29]. Several studies have documented that handwashing with soap may not be viewed as necessary after child cleaning if water or soap are involved. The cleaning process itself could constitute in the caregiver’s mind handwashing as the act its self includes all the same components such as soap, water, and an effort to cleanse. There is a need for more detailed observation on this practice so that a differentiation can be made between child cleaning that used soap and water and child cleaning that did not. 

Collecting observation-based data is labor intensive and subject to a range of biases. The data collection team faced many challenges in ensuring that all relevant information regarding the observed oral contact events was collected. Some oral contact events are infrequent or intermittent and are a part of larger behavior systems. As noted in other studies, observation may have resulted in significant reactivity, changing individual practices to more closely reflect what people know about hygienic behaviors in the home rather than actual practices. However, studies suggest that reactivity to structured observations for hand washing behaviors decreases with the amount of time under observation [30]. Oral contact data skewed heavily female. However, our data suggest that oral contact behaviors in the target age range did not differ by sex of the child. Previous studies included in a meta-analysis of hand-to-mouth frequency data for non-dietary ingestion exposure also noted no difference by child sex [15]. Caregiver observations included only a limited number of critical junctures where handwashing is an important event. Events often occurred in rapid succession proceeded by long periods of inactivity. As such, some were unable to resolve the final sequence of events in 9% of observations. If all of these events happened in the proper sequence, then HWWS at key junctures is still only 14%, below global averages. There were many circumstances where the female head of household agreed to participate in the observation but exogenous events, such as the return of the male head of household, would require the enumerator to terminate the observation. These circumstances resulted in a partial data collection event. The data that was collected in these circumstances was included in the analysis as it was believed that prior to the interruption the observations that were made were representative of the reality of oral contact behavior and caregiving practices in that household. Recommendations for future research in oral contact behavior and caregiver hand hygiene are that study enumerators note the observed household’s access to clean water as this is likely an underlying driver of household hygiene practices. 

## 5. Conclusions

When assessing both infant oral contact events and caregiver hand hygiene practices we have a more comprehensive view of pathogen exposure pathways. Food was the fourth most common type of oral contact event and child feeding was the second most common type of caregiver handwashing key juncture, however handwashing before feeding was only observed in 1% of observations. This indicates that there is a significant opportunity to reduce pathogen exposure for children by addressing the hand hygiene behavior of caregivers before child feedings. Even if our estimates of caregiver handwashing are low, the frequencies are unlikely to be high, highlighting a broad sweeping need for improving caregiver hand hygiene in general. We observed only limited instances of caregiver toileting events and subsequent handwashing, and it is possible handwashing was performed outside of the household. Given the nature of communal latrine infrastructure, targeted observations of handwashing at the site of defecation are necessary in order to more accurately assess hand hygiene in this context. Child toileting and feces handling is a complex system of behaviors and it is recommended that this area of hygiene and sanitation be assessed independently of food preparation and feeding systems so that the necessary amount of detail can be captured. 

## Figures and Tables

**Table 1:** Oral contact observations (*n* = 25).

Total Observation Time *	142:33:00 (hh:mm:ss)
Average Observation Time per Child	05:42:07 (hh:mm:ss)
Index Child Sex Distribution	8 male, 17 female
Index Child Age Distribution	10 infants < 6 months 15 infants 6–9 months

* Observation time was calculated from the first observation to the submission observation log at the end of the observation period.

**Table 2:** Caregiver hand hygiene observations (*n* = 25).

Total Observation Time *	138:11:59 (hh:mm:ss)
Average Observation Time per Caregiver	05:31:37 (hh:mm:ss)
Caregiver Sex Distribution	27 female, 4 male
Caregiver Age Distribution **	Min: 9, Max: 50, Average: 21.8

* Observation time was calculated from the first observation to the submission observation log at the end of the observation period. ** Average caregiver age was calculated based on the information available. There were two observations in which age of the caregiver was not recorded.

**Table 3:** Oral contact events by object.

Oral Contact Object	Average Number of Events per Observation Period	Average Number of Events per Hour
Breast	3.00	0.5
Object	2.49	0.4
Toy	0.60	0.1
Clothing Items	0.48	0.1
Dirt/Soil	0.36	0.1
Phones	0.2	<0.1
Shoes/Sandals	0.16	<0.1
Pacifier	0.16	<0.1
Keys	0.16	<0.1
Furniture/Walls	0.12	<0.1
Bottle	0.05	<0.1
Remote Controller	0.08	<0.1
Cup/Bowl	0.08	<0.1
Spoon/Utensil	0.04	<0.1
Infant’s own hand	2.16	0.4
Foods	1.64	0.3
Cooked Food	1.60	0.3
Uncooked Food	0.04	<0.1
Liquids	0.52	0.1
Water	0.44	0.1
Tea	0.08	<0.1
Other	0.48	<0.1
Wastewater	0.04	<0.1
Medicine	0.12	<0.1
Other	0.32	<0.1
Hands	0.2	<0.1
Caretaker Hand	0.16	<0.1
Other Child Hand	0.04	<0.1
Total	10.56	1.76

**Table 4:** Oral contact event type by age.

Oral Contact Type	3 to <6 Months (*n* = 10)	≥6 to 9 Months (*n* = 15)	Total
Breast	37	38	75
Percent of Observations	49%	51%	28%
Weighted Percent by Age	39%	61%	
Objects	49	15	64
Percent of Observations	77%	23%	24%
Weighted Percent by Age	69%	31%	
Hands Own	29	25	54
Percent of Observations	54%	46%	20%
Weighted Percent by age	44%	56%	
Food	30	11	41
Percent of Observations	73%	27%	16%
Weighted Percent by age	65%	35%	
Liquids	11	2	13
Percent of Observations	85%	15%	5%
Weighted Percent by age	79%	21%	
Other	9	3	12
Percent of Observations	75%	25%	5%
Weighted Percent by age	67%	33%	
Hands Other	2	3	5
Percent of Observations	40%	60%	2%
Weighted Percent by age	31%	69%	
Total	167	97	264
Percent of Observations	63%	38%	100%
Weighted Percent by age	53%	47%	

**Table 5:** Observed critical handwashing junctures.

Juncture	Frequency	Percent
Feeding event	71	70%
Child cleaning	23	23%
Caregiver toilet use	5	5%
Preparing food	2	2%
Total	101	100%

**Table 6:** Caregiver handwashing behavior at critical junctures.

Key Juncture	HWWS	Hand Rinsing	No HW	HWWS; Sequence Not Determined	Total
Before feeding	1 (1%)	0 (0%)	65 (92%)	5 (7%)	71
After child cleaning	2 (9%)	0 (0%)	18 (78%)	3 (13%)	23
After toilet use	2 (40%)	1 (20%)	1 (20%)	1 (20%)	5
Before preparing food	0 (0%)	0 (0%)	1 (50%)	1 (50%)	2
Total	5 (5%)	1 (1%)	85 (84%)	10 (10%)	101

HWWS is handwashing with soap; Hand rinsing is rinsing hands with water without using soap; No HW is no HWWS or rinsing or hand hygiene performed out of sequence; HWWS; sequence not determined are events where caregivers were observed washing hands with soap as part of a larger group of behaviours but proper sequencing of handwashing could not be determined.

## References

[1] Vos T., Allen C., Arora M., Barber R.M., Bhutta Z.A., Brown A., Carter A., Casey D.C., Charlson F.J., Chen A.Z. (2016). Global, regional, and national incidence, prevalence, and years lived with disability for 310 diseases and injuries, 1990–2015: A systematic analysis for the global burden of disease study 2015. Lancet.

[2] Kotloff K.L., Nataro J.P., Blackwelder W.C., Nasrin D., Farag T.H., Panchalingam S., Wu Y., Sow S.O., Sur D., Breiman R.F. (2013). Burden and aetiology of diarrhoeal disease in infants and young children in developing countries (the global enteric multicenter study, gems): A prospective, case-control study. Lancet.

[3] Ngure F.M., Humphrey J.H., Mbuya M.N., Majo F., Mutasa K., Govha M., Mazarura E., Chasekwa B., Prendergast A.J., Curtis V. (2013). Formative research on hygiene behaviors and geophagy among infants and young children and implications of exposure to fecal bacteria. Am. J. Trop. Med. Hyg..

[4] Liu J., Platts-Mills J.A., Juma J., Kabir F., Nkeze J., Okoi C., Operario D.J., Uddin J., Ahmed S., Alonso P.L. (2016). Use of quantitative molecular diagnostic methods to identify causes of diarrhoea in children: A reanalysis of the GEMS case-control study. Lancet.

[5] Fischer Walker C.L., Rudan I., Liu L., Nair H., Theodoratou E., Bhutta Z.A., O’Brien K.L., Campbell H., Black R.E. (2013). Global burden of childhood pneumonia and diarrhoea. Lancet.

[6] Victora C.G., de Onis M., Hallal P.C., Blossner M., Shrimpton R. (2010). Worldwide timing of growth faltering: Revisiting implications for interventions. Pediatrics.

[7] Cumming O., Cairncross S. (2016). Can water, sanitation and hygiene help eliminate stunting? Current evidence and policy implications. Matern. Child Nutr..

[8] Munos M.K., Fischer Walker C.L., Black R.E. (2010). The effect of rotavirus vaccine on diarrhoea mortality. Int. J. Epidemiol..

[9] Prüss-Ustün A., Wolf J., Corvalán C., Neville T., Bos R., Neira M. (2016). Diseases due to unhealthy environments: An updated estimate of the global burden of disease attributable to environmental determinants of health. J. Public Health.

[10] Akland G.G., Pellizzari E.D., Hu Y., Roberds M., Rohrer C.A., Leckie J.O., Berry M.R. (2000). Factors influencing total dietary exposures of young children. J. Expo. Anal. Environ. Epidemiol..

[11] Juberg D.R., Alfano K., Coughlin R.J., Thompson K.M. (2001). An observational study of object mouthing behavior by young children. Pediatrics.

[12] Tulve N.S., Suggs J.C., McCurdy T., Cohen Hubal E.A., Moya J. (2002). Frequency of mouthing behavior in young children. J. Expo. Anal. Environ. Epidemiol..

[13] Kwong L.H., Ercumen A., Pickering A.J., Unicomb L., Davis J., Luby S.P. (2016). Hand- and object-mouthing of rural bangladeshi children 3–18 months old. Int. J. Environ. Res. Public Health.

[14] Mattioli M.C., Boehm A.B., Davis J., Harris A.R., Mrisho M., Pickering A.J. (2014). Enteric pathogens in stored drinking water and on caregiver’s hands in Tanzanian households with and without reported cases of child diarrhea. PLoS ONE.

[15] Xue J., Zartarian V., Moya J., Freeman N., Beamer P., Black K., Tulve N., Shalat S. (2007). A meta-analysis of children’s hand-to-mouth frequency data for estimating nondietary ingestion exposure. Risk Anal..

[16] Aunger R., Schmidt W.P., Ranpura A., Coombes Y. (2010). Three kinds of psychological determinants for hand-washing behaviour in Kenya. Soc. Sci. Med..

[17] Nizame F.A., Leontsini E., Luby S.P., Nuruzzaman M., Parveen S., Winch P.J., Ram P.K., Unicomb L. (2016). Hygiene practices during food preparation in rural Bangladesh: Opportunities to improve the impact of handwashing interventions. Am. J. Trop. Med. Hyg..

[18] Null C., Reese H.E., Moe C.L., Teunis P.F.M., Yakubu H. (2016). Quantifying contact with the environment: Behaviors of young children in Accra, Ghana. Am. J. Trop. Med. Hyg..

[19] Ehiri J.E., Azubuike M.C., Ubbaonu C.N., Anyanwu E.C., Ibe K.M., Ogbonna M.O. (2001). Critical control points of complementary food preparation and handling in eastern Nigeria. Bull. World Health Organ..

[20] Islam M.S., Mahmud Z.H., Gope P.S., Zaman R.U., Hossain Z., Islam M.S., Mondal D., Sharker M.A.Y., Islam K., Jahan H. (2013). Hygiene intervention reduces contamination of weaning food in Bangladesh. Trop. Med. Int. Health.

[21] Touré O., Coulibaly S., Arby A., Maiga F., Cairncross S. (2013). Piloting an intervention to improve microbiological food safety in Peri-Urban Mali. Int. J. Hyg. Environ. Health.

[22] United Nations Human Settlements (2005). Situation Analysis of Informal Settlements in Kisumu.

[23] Scott B.E., Lawson D.W., Curtis V. (2007). Hard to handle: Understanding mothers’ handwashing behaviour in Ghana. Health Policy Plan..

[24] Black K., Shalat S.L., Freeman N.C., Jimenez M., Donnelly K.C., Calvin J.A. (2005). Children’s mouthing and food-handling behavior in an agricultural community on the US/Mexico border. J. Expo. Sci. Environ. Epidemiol..

[25] Mattioli M.C.M., Davis J., Boehm A.B. (2015). Hand-to-mouth contacts result in greater ingestion of feces than dietary water consumption in Tanzania: A quantitative fecal exposure assessment model. Environ. Sci. Technol..

[26] World Health Organization (WHO) (2014). Planning Guide for National Implementation of the Global Strategy for Infant and Young Child Feeding.

[27] Tsou M.-C., Özkaynak H., Beamer P., Dang W., Hsi H.-C., Jiang C.-B., Chien L.-C. (2015). Mouthing activity data for children aged 7 to 35 months in Taiwan. J. Expo. Sci. Environ. Epidemiol..

[28] Curtis V.A., Danquah L.O. (2009). Planned, motivated and habitual hygiene behaviour: An eleven country review. Health Educ. Res..

[29] Nizame F.A., Unicomb L., Sanghvi T., Roy S., Nuruzzaman M., Ghosh P.K., Winch P.J., Luby S.P. (2013). Handwashing before food preparation and child feeding: A missed opportunity for hygiene promotion. Am. J. Trop. Med. Hyg..

[30] Gittelsohn J., Shankar A., West K., Ram R., Gnywali T. (1997). Estimating reactivity in direct observation studies of health behaviors. Hum. Organ..

